# 

**Published:** 1841-01-02

**Authors:** 


### CONTENTS OF VOL. IV.

A

Abdomen, hypertrophy of parietes of the, 481.
Abscess, cases of hepatic, 132, 584; of the ureter, kidney, and lungs, Dr. Stillé's case of, 229.
Abscesses in the brain, cases of, 35; internal, opening into intestinal canal, 356; mammary treated by compression, 498.
Abyssinia, anthelmintics in use in, 497.
Acephalous monster, medico-legal question touching, 724.
Aconitum napellus, cases of poisoning from, 594.
Adipose tumour between the fingers, 686.
Agaric, as a styptic, 9.
Air, secreted from human skin, 596.
Air passages, extraction of a prune-stone from the, 420.
Aleppo button, 442.
Alumina, sulphate of, in inflammations of mucous membranes, 412.
Amaurosis, independent of supra-orbital wounds, 452; cured by iodide of potassium, 835.
Amputation, Dr. Alcock on, 29; statistics of, 189, 498, 707.
Anchylosis, treatment of, 738, 763; of vertebræ, case of, 785.
Andral, M., on the value of chemical and physiological experiments, 98.
Aneurism, varicose, fatal case of, 36; on auscultation in, 220; cases of popliteal, 242, 441; cases of subclavian, 307, 470; of the aorta, 386; of leg, 671.
Anus, artificial, seven cases of, 371; imperforate, Dr. Pancoast's operations for, 392; treatment of pruritus of the, 397, 398; fissure of the, M. Velpeau on, 447; artificial, on the formation of, 527.
Apoplexy, of the lungs, 47; Dr. West's case of, in an infant, 133; case of from fright, 163; on the treatment of, 208.
Aromatic urine, formula for, 20.
Arsenic, M. Orfila on the treatment of cases of poisoning from, 475; in consumption, 496; on the detection of, 658.
Arteries and veins, Joy on diseases of, notice of, 295; cases of ligature of the femoral and external iliac, 373; case of wounded, relieved by the bandage, 535.
Artery, case of rupture of the right coronary, 160; case of rupture of the carotid, 221; pulmonary, cases of absence of, and of irregular disposition of, 225; subclavian, Prof. Gross's case of ligatures of the, 425; wound of the brachial, 665.
Ascites, on the compression of the abdomen in, 143.
Ashmead, Dr. W.'s case of hypertrophy of the heart, 181.
Asphyxia from carbonic acid, 489.
Asthma, cases of thymic, 254, 393.
Azores, a Winter in the, notice of, 538.

B

Bache, Prof. F., notice of introductory lecture by, 823.
Bandaging, new system of, 497.
Bartlett, Prof., notice of introductory lecture by, 823.
Baths, necessity of public, 107; medicated, 202.
Bell, the late John, 225.
Belladonna, ammonia an antidote for, 742.
Berkeley, Dr. C. N., on diseases of the skin, 5, 101.
Betton, Dr. T. F.'s case of strangulated hernia, 214.
Biddle, Dr. J. B.'s case of utero-vaginal rupture, 214; on the use of rhatany in prolapsus ani, leucorrhœa, &c., 293.
Bird, Prof. R. M., notice of introductory lecture by, 701.
Bladder, case of wound of, 145; rupture of, from retention of urine, 625.
Blepharoplastic, Dr. J. M. Warren's operations, 273.
Blood, milky, cause of, 52; colourless globules in the buffy coat of the, 218; arsenic a constituent of the, 224; on the action of some in organic compounds in the, 419; analysis of diseased, 520; blood-discs on the, 419.
Bloodletting, alleged death from excessive, 256.
Brain, effects of contre-coup on the, 382; circulation in the, 796; case of tumor of the, 803.
Brainard, Dr. D.'s case of intra-inguinal hernia, 742.
Bronchocele treated by ligature, 332.
Burns, Dr. R., on the use of iodine in erysipelas, 709.
Burns, soap an application for, 784.

C

Calculus, effects of in the female child, 667.
Callus, case of absorption of during typhus fever, 148.
Cancer of cicatrices, clinical lecture on, 653; of chimney-sweepers, 685.
Cancrum oris, case of, 691.
Carbonic acid gas, as a counter irritant, 19.
Caries of the vertebræ with abscess of the lungs, Dr. J. F. White's case of, 213.
Carotid artery, primitive, case of ligature of, 43.
Carpenter, Dr., in reply to Dr. Paine, 829.
Cataract, operation for, 688.
Cephalalgia, a lotion for, 143; acetic acid in, 556.
Chancres, phagœdenic, iodine in, 497.
Chase, Dr., on the mechanical treatment of deformed feet, 170.
Cheiloplastic operation by Dr. Pancoast, 231.
Chimpanzee, Dr. Hallowell's account of, 88.
Chorda dorsalis, 771.
Chorea, Dr. Prichard's clinical lecture on, 127; on the ammoniuret of copper in, 163, Dr. Webster on, 205.
Cinchona in acute rheumatism, 366.
Club-foot, dissection of a case of, 222.
Coates, Dr. R., on myotomy and tenotomy, 581, 597, 616, 645, 661, 679, 695, 726.
Cod-oil, Mr. Donovan on, 67.
Colhoun, death of Professor Samuel, 239.
Colica pictonum, on the treatment of by warm water, 488.
Combustion, spontaneous, of the human body, 740.
Concretion, a stony, in the human nose, 148.
Convulsions, peculiar case of, 208; cases of puerperal, 223, 592; on the compression of the carotid in, 265.
Cooper, Sir Astley, death of, 217; memoirs of, 274, 313; Sir B. Brodie's eulogium on, 348; case and autopsy of, 364.
Copper, the ammoniuret of, in chorea, 163.
Cornea, on congenital opacity of the, 333, 399; iodine in opacity of the, 498, 583.
Cretinism, on the cure of, 660.
Croup and its cure, 494.
Cryptogamia, developed in vertebræ, 705.
Cubebs and copaiba, M. Ricord on, 416.
Cystecerci, in a tumour, 756.

D

Deformities of the feet, Dr. Chase on the mechanical treatment of, 170.
Dewees, Dr., death of, 326; tribute of respect to, 341.
Digestion, Symonds on diseases of the organs of, 29[illegible].
Dislocation, of the os femoris in an adult female, Dr. Rodgers' case of, 122; of the tibia inwards, case of, 241; forwards of the heads of tibia and fibula, 385; of the cervical vertebræ, 474; congenital of the femur, 498; of the tendon of the biceps, 625; of the ulna, 664; of the wrist, 668; of the clavicle, 802.
Dissection, on the preservation of bodies for, 51.
Drake, Prof., notice of introductory lecture by, 156.
Dropsy, Dr. Prichard's clinical lecture on, 125, on large doses of tartar emetic in the treatment of, 146; after scarlatina, 162.
Dunglison, Prof., notices of introductory lecture by, 702, 789.
Dysentery in Peru, Dr. Smith on, 558.

E

Ear, ulcer of membrana tympani of, cured by alum. 775.
Earle's Visit to European insane asylums, notice of, 251.
Ear, new muscle of the, 338.
Ectropion cured by autoplasty, 498.
Elbow-joint, case of excision of the, 289; M. Roux on resection of the, 450; M. Roux on excision of the, 499.
Embalming, Harlan's Gannal's art of, notice of, 230.
Emoluments, professional, 155.
Emphysema, caused by ulcerated larynx, 836.
Empyema, cases of, 443; cured by paracentesis, 686.
Entozoaria, Dr. Trudeau on the, 405.
Epidemics of 1841 in Philadelphia, 183, 453, 698.
Epilepsy, case of, 477.
Ergot, Dr. Beck on the abuse of, 325; on the use of, 639.
Erysipelas, M. M. Vidal, Chelius, and Liston on, 430.
Esophagus, case of scirrhus of the, 816.
Esquirol, biographical sketch of, 329.
Ethics, medical, remarks on a case of, 619.
Evidence, medical, character of, 93.
Experiments on the bodies of criminals, 107.
Eye, action of oblique muscles of the, 411; on improvement of refractive power of the, by myotomy, 500: on aponeuroses and muscles of, 526; diseases of, cured by galvanism, 703; hairs within the, 752; action of hydrocyanic acid on the, 814.
Eyelids, horny excrescences on the, 496.

F

Face, new arrangement of muscles of the, 338.
Fallopian pregnancy, case of, 638.
Fever, remittent, Stewardson on, review of, 235; Dr. Stratton on the lake-fever of Canada, 346; epidemic of in the West, 503; on the forms of remittent and intermittent, 535.
Fistula, case of cœcal, 162.
Fœtus, case of injury to the head of, from deformity of pelvis, 15; communication of intermittent fever to, from mother, 494; removal of through vagina in a case of extra-uterine fœtation, 815.
Fractures of the neck of the femur, 159; Dr. Norris' statistics of, 267; Dr. Fricke's statistics of, 296; case of ununited, of the humerus, 500; case of ununited, treated by seaton, 549; of the neck of the thigh bone within capsular ligament, 625; of the internal cord of the humerus, 665; immovable apparatus for, 724; partial, of the radius, 731.
Fungus hæmatodes, Dr. Upshur's cases of, 667; cases of, 723, 826.

G

Gall-bladder, fibres in the walls of the, 420.
Galvanic experiments on the body of a criminal, 58, 76.
Galvanism, Dr. Fowler on, 562.
Gangrene, of the lungs, Dr. Gerhard, 21; case of, 51; semile, lecture on, 316.
Gastrotomy, and gastro-hysterotomy, Dr. Warrington on, 165; cases of, 413, 499; in a cow, for cure of intestinal invagination, 524.
Gelatine, on the oxidation of, 755.
Gerhard, Dr. lectures by, on gangrene of the, lungs, 21; on the tuberculous phthisis, 37, 69, 85, 116, 197, 245, 261, 357; on tubercles of the bronchial glands, 389; on pulmonary hæmorrhage, 421; on pericarditis, 485; on diseases of the heart, 613, 693, 725, 741, 773, 805, 821.
Gibson, Prof. W's. Rambles in Europe, notice of, 199; notice of introductory lecture by, 757; operation by, for anchylosis, 763.
Glanders, case of development of, 144; in man, 622.
Goitre cured by ligature, 627.
Gonorrhœa, chloride of zinc in, 415.
Gout, cured by the electric shock, 297.
Green, prof. Jacob, death of, 94.
Guerin, M., on the subcutaneous section of muscles, 144; on a subcutaneous operation for strangulated hernia, 755.

H.

Hæmorrhage, fatal case of, in an infant, 243, of the lungs, Dr. Gerhard on, 421, cases of secondary, 466, 467; new treatment of puerperal, 499, 589.
Hæmitis, M. Piorry on, 63.
Hæmorrhoidal tumours, reduction of 452.
Hamilton, prof. F. H.; notice of introductory lecture by, 62.
Hanging, cases of, death by, 736.
Harrison, case of the late President William Henry, 309.
Head, case of, severe injury of the, 240.
Heart, experiments on the motions and sounds of, 10; case of hypertrophy of the, 181; rare malformation of the, 321; case of wound of the, 451; Dr. Gerhard on diseases of the, 613, 693, 725, 741, 773, 805, 821; cases of diseased, 733.
Helmuth, Dr. on corrosive sublimate in dysentery, 533; on iodine in opacity of the cornea, 583.
Hermaphrodite, anatomical description of, 232
Hernia, M. Velpeau, on a new operation for the radical cure of, 145; Dr. Betton's case of strangulated scrotal, 214; case of congenital of the heart and stomach, 226; Dr. Stillé's case of strangulated scrotal, 230; Mr. S. Cooper's lecture on strangulated femoral, 379; of the uterus, 499; case of diaphragmatic, 533; remarkable cases of, 667, 724; fatal cases of inguinal, 694, 742.
Hope, sketch of the late Dr., 505.
Horner, prof. W. E's. introductory lecture, notice of, 743.
Hospitals, of Paris, Dr. Linton's account of, 77; report of Massachusetts general hospital, 156; of Metz, 350; in China, 508: Foundling, of Petersburg, 523.
Humerus, excision of the head of the, 307, 739.
Hydatid tumour, in the loins, 684; in the breast, 685.
Hydrocele, on tincture of iodine in, 409; cases of, 550, 551, 789; new treatment of, 604.
Hydrocephalus, case of acute, cured, 260; cold affusion in 735.
Hydrophobia, Dr. Triberti on, 146; experimental researches on the mode of transmission of, 147.
Hydrosudopathy, 76.
Hygiene, notice of journal of, 710.
Hyperoperating, 730.

I.

India, practice in, 600; vaccination in, 601.
Inflammations, abdominal, diagnosis of, 504.
Intussusception of the ileum, 756.
Iron, lactate of, 52; ioduret of, for syphilitic ulcers, 84; ioduret of, mode of preparing, 212; sesquioxide of, purification of, 223, 594; an antidote for Scheele's Green, 324.
Iris, physiology of the, 494; injections of the, 740.
Inoculation of the cow with variolous matter, 782.
Insane poor, 27; necessity of asylum for, 27; Connecticut retreat for, 28; Pepperell institution for, 62; organization of Pennsylvania asylum for, 501.
Intestine, wound of, 296.
Iodine, in frostbite, Dr. Wright on the use of, 181; inhalation of, in consumption, 478, 556; in phagœdenic chancers, 497; in opacity of of the cornea. 498; in erysipelas, 709.
Itch, formula for, 165; mode of treating, at Berlin, 515.

J.

Jackson, prof. S. notices of introductory lecture by, 715, 758.
Jaundice, cases of, caused by aneurism of mesenteric artery, 625.
Johnson's syrup, 276.
Johnston, Dr. W. Poyntell, on the use of rhatany in fissure and prolapsus of the anus, &c., 293.
Joints, Brodie on diseases of the, review of, 153, 216; on the removal of foreign bodies in the, by subcutaneous incisions, 415.
Journals, medical, on the increase of 43; N. York Medical Gazette, 517; Western and Southern medical, 758.

K.

Kidney, case of granular disease of the, 831.
Kidneys, great developement of, 836.
Kiesteine, 276.
Knee-joint, amputation at the, 824.
Kreosote in affections of the eye, 668.
Kyanised timber, 414.

L.

Laryngitis, acute, Dr. Prichard's clinical lecture on, 126.
Laryngotomy, operations of, 520.
Lead, acetate of, poisoning by, 496.
Leeches poisoned by blood of the sick, 495; on the reproduction of, 737.
Le Monnier on obstetrics, review of, 120.
Ligament, round, of the hip-joint, 799.
Ligature, effect of, on femoral artery, 626.
Lingualis muscle, 482.
Lip, Dr. Townsend's cases of warty excrescences of the, 454.
Lithotomy, lecture by Mr. Bransby Cooper on, 326; statistics of, in Naples, 515.
Lithotrity, antiquity of, 692.
Louis, on typhoid fever, review of, 134, 149.
Luscitas spastica, cases of, 621, 641.
Lymphadenitis, case of idiopathic, 142.
Lymphatics of the brain, 222.

M.

Magnetism, Animal, Dr. R. Coates on, 716, 790, 808.
Malpractice, case of, at Cortlandville, 712, 766.
Malta, as a residence for invalids, 539.
Mania, on bleeding in, 418.
Marine invertebrata, on the temperature of, 740.
Mastodon, a new specimen of, 731.
McClellan, Prof. George, operation by, for the removal of spina ventosa of the skull, 44.
McClintock, Dr, James, notice of introductory lecture by, 62.
McNeven, death of Dr., 517.
Meigs, Prof. notice of introductory lecture by, 777.
Mercer Dr. J. C.'s case of tuberculous disease, 249, 373.
Mercury, bichloride of, in dysentery, Dr. Helmuth on, 533.
Meteorological registers, 100, 164, 244, 308, 372.
Menstruation, statistics of, 644.
Mildew, remarks on, 185.
Milk, on the microscopic constitution of, 179, 521.
Milk sickness, Dr. Graff on, 280; in Tennessee, 502.
Miller, Prof. Thomas, notice of introductory lecture by, 24; case of President Harrison by, 209.
Miner, death of Dr. Thomas, 312.
Molluscum contagiosum, 608.
Morphia, chemico-legal researches on, 558; case of poisoning from endermic use of, 695.
Mortality, bills of, 98, 114, 122, 140, 158, 180, 188, 205, 218, 240, 254, 272, 292, 296, 313, 325, 341, 364, 388, 408, 425, 452, 455, 484, 500, 516, 532, 535, 551, 596, 600, 628, 629, 660, 663, 592, 708, 722, 631, 750, 772, 788, 804, 820, 836.
Mucous membranes, Dr. Hodgkin on diseases of the, 442.
Murrain of cattle, 683.
Muscæ volitantes, 468.
Mütter, Prof. notice of introductory lecture by, 716.
Myotomy, impropriety of, for cure of curvature of the spine, 524; of the pronator muscles of the hand and fingers, 548, Dr. R. Coates on, 581, 597, 616, 645, 661, 679, 695, 726; Mr. Liston's operation for, 684.

N.

Naval surgeons passed in 1841, 454.
Nerves, on the anastomoses of, 509; on the motor influences of the cerebral and cervical, 511; on electrical currents in, 519; functions of the roots of the, 524.
Neuralgia, case of, 356; facialis, case of, 517; new mode of treating functional, 546.
Nipples, artificial, of ivory, 116.
Non-vascularity of certain animal tissues, 689.

O.

Obstetric reports, Dr. Warrington's, 73, 294.
Ontology, remarks on, 61.
Ophthalmia, purulent of almshouses, Dr. Morrell on, 109; neonatorum, Dr. Cederschjöld on, 222.
Orbit, anatomy and physiology of certain structures in the, 691.
Over-eating, effects of, 180.
Opii tinctura ammoniata, 493.
Orchitis, new treatment for, 500.
Oxalic acid, in inflammations of mucous membranes, 627.
Ozœna, on the cure of, 753.

P.

Paine's Commentaries, the British and Foreign Review on, 407.
Palm, wound of the, 670.
Pancoast, Prof. Joseph's rhinoplastic and cheiloplastic operations, 231; operations for strabismus, 390; operation for imperforate anus, 392; amputation by, at the knee-joint, 824.
Paralysis of the fifth pair, cured by galvanism, 420; from visceral disorders, 750.
Parrish, Dr., notice of Prof. Wood's memoir of, 25.
Parturition, excitement of premature, 17.
Paullinia, Dr. Gavelle on, 243.
Pelvis, case of tumour of the, 410.
Perception, case of suspended, 23.
Pericarditis, Dr. Gerhard on, 485; cured by paracentesis, 524.

Pernio, effects of, 396.
Phallus impudicus, 687.
Phlebitis, remarkable case of, 376.
Phthisis, tuberculous, Dr. Gerhard on, 37, 69, 85, 116, 197, 245, 261, 357 ; on inhalation of iodine and conium in, 479 ; arsenic in, 496.
Pinel, lawsuit for the skull of, 212.
Pitch against piles, 243.
Placenta, Dr. Wedderburn's case of fatty degeneration of, 72 ; on the structure of, 417.
Platinum, therapeutic effects of, 212, 412.
Plica polonica, pathology of, 495.
Pneumonia, statistics of, 46; Dr. Sale's case of, 501.
Potash, nitrate of, in rheumatism, 495.
Potassium, iodide of, in syphilis, 224 ; in amaurosis, 835.
Pregnancies occurring during amenorrhœa, 224.
Prize questions, medical, for 1841, 29.
Pseudarthrosis, on the treatment of, 532.
Pseudo-membranous inflammation of pharynx, contagiousness of, 753.
Ptyalism, best produced by small doses of mercury, 670, 819.
Pulse, daily revolutions of the, 223 ; case of slow, 355.
Pylorus, scirrhus of the, 834.

Q.

Quackery, 830.
Quadruplets, case of, 226.
Quinine, Sulphate of, in enlargement of the spleen, and dropsies after agues, 68 ; on the hydrocyanoferrate of, 132 ; a poison for rabbits, 276.

R.

Rachitis, M. Guérin on, 113.
Rectum, case of removal of, 527.
Reform, medical, editorial comments on, 440, 453, 469.
Retention of urine, case of, 270.
Re-vaccination, 767.
Rhatany, Drs. Johnston and Biddle, on the use of, in fissure and prolapsus of the anus, leucorrhœa, &c., 293 ; formula for solution of, 341.
Rheumatism, Dr. Washington's case of remarkable effects of, 123 ; on the use of cinchona in acute, 366 ; nitrate of potash in, 495.
Rhinoplastic operation by Dr. Pancoast, 231.
Rigidity, case of early cadaveric, 403.
Rupture of the liver and spleen, case of, 551 ; of the rectus femoris, 804.

S.

Sale, Dr. R. A.'s case of pleuro-pneumonia, 501.
Scarlatina, Dr. Bornell on the treatment of, by stimulants, 94.
Schools, medical, notices of—of Harvard University, 62 ; Geneva, 77 ; of Albany, 94 ; of the University of New York, 97 ; Louisville Medical Institute, 156 : University of Edinburgh, 227 ; Jefferson Medical, 239 ; summary of, 239 ; Transylvania, 240 ; of Hamburgh, 424 ; College of Physicians and Surgeons of New York, 794.
Scirrhus of the brain, case of, 222.
Shoulder-joint, case of amputation of, 226.
Silver, on the oxide of, 629 ; nitrate of, in enteritis, 817.
Singultus, case of, cured by a seton, 148.
Skin, Dr. Berkeley on diseases of the, 5, 101.
Skulls, of the ancient Peruvians and Mexicans, Prof. Morton on, 719.
Society, Philadelphia Medical, officers of for 1841, 29 ; Medical, of Hamburgh, 424.
Spleen, engorgements of, mode of removing, 144.
Spina bifida, on the radical cure of, 770.
Spina ventosa of the skull, Dr. McClellan's operation for the removal of, 44.
Spinal marrow, on the functions of different portions of, 120, 519, 524 ; cases of wound of, 123, 413.
Spine, on distortions of, from enlargement of the abdomen, 353.
Stammering, remarks on, 114 ; operations for the cure of, 342, 392, 407, 676 ; hæmorrhage after operation for, 497 ; Dieffenbach on, 516.
Stokes on the theory and practice of physic, review of, 75.
Stomach, healthy appearance of internal surface of the, 494.
Strabismus, Mr. Guthrie on, 81 ; Dr. Ingalls' claims to the origination of the operation for the cure of, 119 ; dissection of a case after the operation for, 260 ; Prof. Pancoast on the operation for, 390 ; Dr. Dix on, 437.
Strangulated intestine, case of, 20.
Sudden death, causes of, 128.
Syphilis, iodide of iron in the treatment of, 84; iodide of potash in, 224 ; remarkable case of secondary, 420 ; in pregnant females, 706 ; ulcers from, 729.

T.

Talipes, on the operation for, 400.
Taxis, rupture of the intestine during, 637.
Tenotomy, Dr. R. Coates on, 581, 597, 616, 645, 661, 679, 695, 726.
Testicle, case of congenital absence of one, 73; case of cystic disease of the, 465.
Tetanus, traumatic, Dr. Bowen on the treatment of, by tobacco enemata, 204; Dr. Watson on the treatment of, 530.
Thorax, mode of evacuating effusions into the, 325.
Tickner, Dr., notice of address by, 121.
Tobacco, case of poisoning from, 476.
Torticollis, on a new species of, 145 ; operation for, 322.
Transactions of Royal Medical and Chirurgical Society, 625, 795.
Trigeminus nerve, case of irritation of, 140.
Trudeau, Dr., on the compression of the carotid in convulsions, 265 ; on entozoaria, 405; operations for stammering, 392, 407.

Tubercles of the larynx and fauces, 161 ; of the bronchial glands, Dr. Gerhard on, 389 ; in the cerebellum, 462.
Tubercular disease, Dr. Mercer's case of, 249, 373.
Tweedie's Practical Medicine, reviews of, 103, 463.
Typhoid fever, review of Louis on, 134, 149.

U.

Umbilical cord, 477, 784.
Upshur, Dr. A. W., on yellow fever, 277, 361; cases of fungus hæmatodes by, 677.
Urethra, stricture of, on dilatation by fluid pressure in, 544 ; extraction of a pin from, 666.
Urethro-plastic operation of Ricord, 753.
Urinary organs, Christison on diseases of, notice of, 295.
Urine, biphosphate of magnesia and ammonia in the, 409 ; antimony in the, 406.
Uterine organs, Ferguson and Simpson on diseases of, notice of, 295.
Utero-vaginal rupture, Dr. Biddle's case of, 214 ; Dr. Wilson's case of, 263.
Uterus, Dr. Warrington on the rupture of, 53; Dr. Post's case of sloughing of, 123 ; danger of injecting fluids into the; nerves of the, 435 ; amputation of the neck of the, followed by pregnancy, 455, 460 ; case of procidentia of, successfully treated, 517 ; unimpregnated case of retroversion of the, 520 ; extraction of a foreign body from the, 596 ; extirpation of the neck of the, 644 ; spurious labour pains, 691 ; on encysted tubercles in the, 755 ; case of rupture of the, 811.

V.

Vaccination, Dr. Gregory on, 298 ; in India, 504.
Vagina, case of imperforate, 115.
Varicocele, radical cure of by invagination, 416.
Variola, case of posthumous, 144 ; scars of, prevented by mercurial plaster, 271, 557 ; epidemic of, in Philadelphia in 1841, 295 ; on the treatment of by the ectrotic plan, 299; identity of, with cow pox, 627.
Vectis, on the use of the, 633, 648.
Venesection, wound of the brachial artery in, 699.
Viscera, case of transposition of, 224.
Vitriol, elixir of, 307.

W.

Warrington, Dr. on rupture of the uterus, 53 ; obstetric reports by, 73, 294 ; on gastrotomy, 165, 487, 682.
Wedderburn, Dr. A. J.'s case of diseased placenta, 72.
Weights, French decimal system of, 66.
West, Dr. F.'s case of apoplexy in an infant, 133.
White, Dr. J. F.'s case of caries of the vertebræ, 213.
White swelling, on the treatment of, 417 ; nitrate of silver in, 753.
Wilson, Dr. J. F.'s case of utero-vaginal rupture, 263.
Wood, Prof., notice of introductory lecture by, 703.
Wright, Dr. D. M., on iodine in frost bite, 181.
Wounds, lacerated, cases of, 730, 833.

Y.

Yellow fever, Dr. Upshur on, 277, 361.

Z.

Zinc, chloride of, in gonorrhœa, 415.

Zoospermata, 416.

